# Renal Impairment After Cardiac Surgery: Risk Factors, Outcome and Cost Effectiveness

**DOI:** 10.7759/cureus.11694

**Published:** 2020-11-25

**Authors:** Yasser A Elghoneimy, Abdulaziz AL Qahtani, Sultan A Almontasheri, Yousef Tawhari, Mohammed Alshehri, Abdulaziz H Alshahrani, Saad Almashi

**Affiliations:** 1 Department of Cardiac Surgery, King Fahad University Hospital, Al Khobar, SAU; 2 Department of General Surgery, Imam Abdulrahman Bin Faisal University, Dammam, SAU

**Keywords:** acute kidney injury, cardiac surgery, post cardiac surgery, financial burden, risk factors

## Abstract

Introduction

Acute kidney injury (AKI) is considered one of the serious complications in the medical field. It has a large impact on patients' life medically, socially and economically. It also has a financial burden on governments and hospitals regardless of which part of the world is considered. On the other hand, AKI is a common complication of cardiac surgery, which alone has a tremendous burden and implications on patients and governments. In this study, we will discuss the various risk factors, outcomes and financial burden of renal impairment associated with cardiac surgery.

Methods

This is a retrospective case-control study, which included 144 adult patients who underwent open cardiac surgical procedures at King Fahad University Hospital in the Eastern Province of Saudi Arabia over a period of five years from January 2015 till the end of December 2019. We included all types of cardiac surgeries performed such as coronary artery bypass grafting (CABG), valve surgery and aortic dissection repair and excluded patients with end-stage renal disease (ESRD) requiring dialysis preoperatively and pediatric patients. Two control groups were defined, those who developed renal impairment (group A) and those who did not develop it (group B).

Results

The mean age of the patients was 58.59 ± 12.6 years (range: 42 to 77 years). Mean serum creatinine level in the postoperative period was 1.95 ± 1.5 mg/dL in group A compared to group B of 1.0 ± 0.32 mg/dL (P-value<0.01). Mean serum blood urea nitrogen (BUN) in group A was 26.45 ± 19.9 mg/dL compared to group B of 16.79 ± 16.2 mg/dL in group B (P-value < 0.01). Diabetic were more likely to develop renal impairment than non-diabetic (P-value = 0.049, OR 2.73; 95% CI: 0.97-7.66). Obese patients were two times more likely to develop renal impairment than non-obese (P-value = 0.056, OR 2.6; 95% CI: 0.94-7.1). The average cost for each patient with renal impairment who required dialysis was 110,000 Saudi Riyal (~ 29,000 $) compared to other patients.

Conclusion

Serum creatinine, BUN, diabetes and obesity are strong indicators in developing AKI in cardiac surgery. In addition, the financial burden was almost doubled in patients developing AKI.

## Introduction

Acute renal failure or acute kidney injury (AKI) following cardiac procedure is defined according to the Kidney Disease: Improving Global Outcomes (KDIGO) as decrease in urine output and increase in serum creatinine [1]. It is a common complication after cardiac surgery due to the rapid deterioration of the glomerular filtration rate (GFR) [2]. Surgery-associated acute kidney injury (SA-AKI) is one of the serious complications of cardiac surgery with a prevalence rate of up to 30% [2]. It is believed that SA-AKI plays a sole factor in increasing mortality and morbidity rates among cardiac surgery patients [2]. Similarly, mortality rates of the intensive care unit (ICU) patients by itself have a great impact on kidney function, adding the burden of SA-AKI increases it further [2]. Many risk factors have been associated with increased incidence of SA-AKI including old age, females, preoperative cardiac dysfunction, emergent surgery, peripheral arterial disease, insulin-dependent diabetes and preoperative renal dysfunction [2]. To this date, many modules have been proposed to predict the need for dialysis, however, they are yet to be considered of good use in predicting SA-AKI in cardiac surgeries. Such scores include the Cleveland Clinic Score (TCCS) [3], Mehta score [4] and Simplified Renal Index (SRI) score [4]. Generally, as the case with all chronic illnesses, AKI after cardiac surgery has an economic and social burden on the hospital, government and patients. In the United States, the society of thoracic surgeons estimated the overall national financial index of patients with AKI as a complication of thoracic surgery to be approximately one billion dollars with a mean cost of 77 thousand dollars per patient [5]. In Saudi Arabia, the latest numbers in 1990 estimated that there are more than 2,000 open heart surgery is performed yearly in the kingdom [6]. Therefore, we aim to identify and report the different risk factors associated with developing AKI after cardiac surgery as well as any associated financial burden and outcome.

## Materials and methods

This is a retrospective case-control study, which included 144 adult patients who underwent open cardiac surgical procedures at King Fahad University Hospital in the Eastern Province of Saudi Arabia over a period of five years from January 2015 till the end of December 2019. The study was approved by the Institutional Review Board (IRB) with approval number: IRB-UGS-2019-01-369. We included all types of cardiac surgeries performed such as coronary artery bypass grafting (CABG), valve surgery and aortic dissection repair. Meanwhile, we excluded patients with end-stage renal disease (ESRD) requiring dialysis preoperatively and pediatric patients. Data collection was collected from three time points: preoperative assessment, operative data and postoperative outcomes. Afterwards, we divided the patients into two control groups. Those who developed renal impairment (Group A) against those who did not develop it (Group B). Renal impairment was defined as a decrease in urine output (< than 0.15 ml/kg per hour to six hours) and an increase in serum creatinine of more than 1.5X over seven-day duration [1]. Statistical package for social sciences (SPSS v.23 IBM Corp., Armonk, NY) was used for data entry and analysis. Average values, standard deviation, frequencies and percentages were used to present descriptive statistics. In inferential statistics, chi-square test and odd ratios were used to analyze the post-operative complication. Pre-operative parameters were categorized according to the type of surgery. Two independent sample T-test was used to test post-operative complication with continuous variables and the level of significance was set at 0.05.

## Results

A total number of 144 patients were included in the study, 103 males (71.5%) and 41 (28.5%) females. The mean age of the patients was 58.59 ± 12.6 with the youngest included patient was 42 years old and the oldest was 77 years old. Regarding comorbidities, most of the patients had hypertension 89 (61.8%) followed by diabetes in 44 (30.6%) and chronic obesity was seen in 44 (30.6%) while the least reported comorbidities were hypercholesterolemia 16 (11.1%) and smoking 16 (11.1%) (Table 1). Meanwhile, the most performed surgical procedure was CABG 105 (72.9%) followed by valve surgery in 30 patients (20.8%) and others 9 (6.3%).

**Table 1:** Preoperative associated co-morbid conditions in all patient

Parameters	Present Number (%)	Absent Number (%)
Chronic Obesity	44 (30.6)	100 (69.4)
Smoker	16 (11.1)	128 (88.9)
Diabetes	44 (30.6)	93 (64.6)
Hypercholesterolemia	16 (11.1)	127 (88.2)
Hypertension	89 (61.8)	54 (37.5)

Preoperative baseline renal function showed that the mean serum creatinine level was 1.12 ± 0.67 mg/dL, serum sodium 139.69 ± 5.37 mEq/L, BUN 17.99 ± 9.9 mg/dL and potassium level 4.55 ± 3.87 mEq/L. Postoperatively, mean serum creatinine in group A was 1.95 ± 1.5 mg/dL with statistical significance of P-value < 0.01 compared to group B which was 1.0 ± 0.32 mg/dL. Moreover, Serum BUN had a mean value of 26.45 ± 19.9 mg/dL in group A compared to a mean value of 16.79 ± 16.2 mg/dL in group B and comparison analysis showed it had a statistical significance of P-value < 0.01.

Among renal impairment risk factors, diabetic patients were two times more likely to develop renal impairment than non-diabetic with a significant P-value = 0.049 (OR 2.73; 95% CI: 0.97-7.66). Furthermore, obesity was also found to be significantly associated with renal impairment, where the chance of renal impairment was two times higher with a P-value = 0.056 (OR 2.6; 95% CI: 0.94-7.1). On the other hand, odds of hypertensive patients developing renal impairment was also found to be 2.3 more likely to happen in comparison to non-hypertensive patients, however, no statistical significance was found. Lastly, hypercholesterolemia had no effect on patients (Table 2).

**Table 2:** *Statistically significant at 0.05 level of significance

Pre-operative parameters		Post-operative complication		P-value	Odd Ratio (95% CI)
		Renal impairment	Normal		
Chronic Obesity	Yes	9 (20.5%)	35 (79.5%)	0.056	2.6(0.94-7.1)
	No	9 (9.0%)	91(91.0%)		
Smoker	Yes	2 (12.5%)	14(87.5%)	1.00	1.0(0.21-4.8)
	No	16 (12.5%)	112 (87.5%)		
Diabetes	Yes	9 (20.5%)	35 (79.5%)	0.049^*^	2.73(0.97-7.66)
	No	8 (8.6%)	85 (91.4%)		
Hypercholesterolemia	Yes	2 (12.5%)	14 (87.5%)	0.991	0.99(0.21-4.77)
	No	16 (12.6%)	111 (87.4%)		
Hypertension	Yes	14 (15.7%)	75 (84.3%)	0.146	2.33(0.73-7.5)
	No	4 (7.4%)	50 (92.6%)		

Regarding surgical procedures, neither CABG nor valve surgery had any significant association with renal impairment as an outcome. However, 15 out of 18 patients that had renal impairment had CABG, where odds of complication in CABG was two times higher than other procedures (P-value = 0.288) (Table 3). Meanwhile, patients in group A had a prolonged ICU stay compared to group B. The mean ICU length of stays for group A was 15.8 ± 2 days compared to 9.98 ± 1 day for group B. Six of the patients who had renal impairment (33.3%) required Continuous Veno-Venous Hemodialysis (CVVHD) and the dialysis mean duration was 6 ± 2.0 days.

**Table 3:** Association of post-operative complication with type of surgical procedure

Surgical Procedure		Post-operative complication		P-value	Odd Ratio (95% CI)
		Yes (Renal Impairment)	No		
Coronary Artery Bypass Grafting (CABG)	Yes	15 (14.3%)	90 (85.7%)	0.288	2.0(0.55-7.3)
	No	3 (7.7%)	36 (92.3%)		
Valve surgery	Yes	3 (10.0%)	27 (90.0%)	0.64	0.733(0.2-2.72)
	No	15 (13.2%)	99 (86.8%)		

Finally, the average cost for each patient with renal impairment requiring dialysis was 110,000 Saudi Riyal (~ 29,000 $). The finance calculation included ICU stay, cost of dialysis, medication and lab investigation. In comparison to patients who did not require analysis, the average cost was almost lowered by half around 50,000 SR. Nonetheless, these costs did not count medical staff and hospital administration fees.

## Discussion

The prevalence of renal impairment or AKI after cardiac surgical procedures varies between 5% and 20% [2]. Incidence of kidney injury is 1% to 2% of patients requiring dialysis and undergoing cardiac surgery [7,8]. Palamuthusingam et al. analyzed 11,000 patients from 15 studies who underwent cardiac surgery and found that they were four times more likely to die with complication of AKI (OR 4.23, 95% CI: 3.21-5.56). In addition, Santos et al. in a prospective study in 2016 reported that diabetic CABG patients were also reported to have an increased incidence of AKI up to 5.6% and a higher mortality rate when compared to non-diabetics [9]. In comparison to our findings, we found that diabetic patients are more likely to develop AKI 2.3 more times than non-diabetics. However, Santos et al. correlated the AKI to many predisposing factors including infection was more significantly associated with AKI due to host defense impairment from high blood sugar, increased stress and decreased cellular immunity [9].

Hypertension and AKI can both be primary or secondary to one another. Pathophysiologically, AKI developing in hypertensive patients are most likely due to structural changes in the renal arterioles affecting the renal hemodynamics [10]. In 2018, Barkhordari et al. did a cross-sectional study on 3473 patients and showed that hypertension among other risk factors were significantly associated with a higher rate of AKI after CABG surgery [11]. In the same context, Mitrev et al. found that the effect of increased pulse pressure intraoperatively was independently and significantly associated with postoperative AKI with every 1-mmHg increase in pulse pressure a 1.07% increase rate of AKI was noted [12]. Increased pulse pressure is one of the predisposing risk factors in developing hypertension, myocardial infarction and congestive heart failure even with antihypertensive and cardiac medications [13].

In general, renal function can be measured in various ways in the lab which includes creatinine, GFR or BUN. Therefore, such investigational predictive value cannot be sufficient by itself because it usually takes 24 to 72 hours for the deterioration to be measured in the lab [14]. However, creatinine clearance measurement can be more sensitive and effective if utilized among other factors. Collectively, all biomarkers combined can provide us with better predictive factors. In our study, serum creatinine mean value was 1.95 ± 1.5 mg/dL postoperatively and was a significant factor in AKI patients (P < 0.001). Therefore, studying all renal biomarkers together might have a value in predicting AKI before cardiac surgeries, especially in diabetic patients [14].

Harky et al. stated that during cardiac surgery several pathophysiological factors taking place on a microvascular level affect the renal function. These include hypoperfusion, renal artheroemobolism, activation of complement and inflammatory mediators due to surgical stress and neurohormonal factors. Meanwhile, preoperatively, females are more susceptible to develop AKI post-cardiac surgery compared to men [15]. In our study, gender was not further studied due to the relatively low number of cases.

AKI requiring dialysis have an increased risk of mortality of 1.2%-3.0% post-cardiac surgery. Furthermore, post-cardiac surgery AKI is the most common cause of kidney injury among ICU admissions [16]. Since cardiac surgery patients are administered numerous drugs, some of the intraoperative as well as postoperative can be nephrotoxic. Nonetheless, these patients are prone to infections and are usually are started on antibiotics therapy of which some might have a damaging effect on the renal function [16]. It is also noted that the lengthier the operation is, the more likely patients would develop AKI post-surgery during their ICU stay. Moreover, the length of stay in the ICU has an increased risk factor in developing AKI and an even increased risk when combined with cardiac surgeries [16]. Our included patients were more likely to develop AKI in CABG procedures.

In our study, the financial burden almost doubled when we compared patients not requiring dialysis with those who did need it due to AKI. Developing predictive scores and continuous studies are needed in order to lower mortality rates among cardiac surgery patients as well as decrease the financial burden governments and individuals have to carry moving forward. Limitation of our study includes the relatively low sample of female gender. Also, perioperative infections were not considered in the study. Different cardiac procedures and the length of operation were not explored due to a lack of sufficient data. Also, the types of medication taken and dosage was not considered as we focused on the indicators, risk factors and financial burden in detail. Lastly, creatinine clearance was not utilized by the physicians in their reports in determining the renal function.

## Conclusions

Serum creatinine, BUN, diabetes, obesity, prolonged ICU length of stay and type of cardiac procedure (i.e., CABG) are strong indicators for developing acute kidney injury in cardiac surgery. In addition, the financial burden was almost doubled in patients developing renal impairment. Studying and developing a prediction module to minimize risk factor is crucial to lower the incidence of renal impairment post-cardiac surgery and decrease the financial burden on the governments around the world.

## References

[1] Levey AS, Eckardt K-U, Tsukamoto Y (2005). Definition and classification of chronic kidney disease: a position statement from Kidney Disease: Improving Global Outcomes (KDIGO). Kidney International.

[2] Vives M, Hernandez A, Parramon F (2019). Acute kidney injury after cardiac surgery: prevalence, impact and management challenges. Int J Nephrol Renovasc Dis.

[3] Thakar CV, Arrigain S, Worley S (2005). A clinical score to predict acute renal failure after cardiac surgery. J Am Soc Nephrol.

[4] Mehta RH, Grab JD, O'Brien SM (2006). Bedside tool for predicting the risk of postoperative dialysis in patients undergoing cardiac surgery. Circulation.

[5] Alshaikh HN, Katz NM, Gani F (2018). Financial impact of acute kidney injury after cardiac operations in the United States. Ann Thorac Surg.

[6] Duran C (1990). Cardiac surgery in the Kingdom of Saudi Arabia. Ann Saudi Med.

[7] Mariscalco G, Lorusso R, Dominici C (2011). Acute kidney injury: a relevant complication after cardiac surgery. Ann Thorac Surg.

[8] Palamuthusingam D, Nadarajah A, Pascoe EM (2020). Postoperative mortality in patients on chronic dialysis following elective surgery: a systematic review and meta-analysis. PLoS One.

[9] Santos KAQ, Berto B, Sousa AG, Costa FAAd (2016). Prognosis and complications of diabetic patients undergoing isolated coronary artery bypass surgery. Braz J Cardiovasc Surg.

[10] Harty J (2014). Prevention and management of acute kidney injury. Ulster Med J.

[11] Barkhordari K, Fakhre Yasseri AM, Yousefshahi F, Shafiee A (2018). Risk factors for acute kidney injury in coronary artery bypass graft surgery patients based on the Acute Kidney Injury Network criteria. J Tehran Heart Center.

[12] Mitrev L, Speich KG, Ng S (2019). Elevated pulse pressure in anesthetized subjects before cardiopulmonary bypass is associated strongly with postoperative acute kidney injury stage. J Cardiothorac Vasc Anesth.

[13] Safar ME (2001). Systolic blood pressure, pulse pressure and arterial stiffness as cardiovascular risk factors. Curr Opin Nephrol Hypertens.

[14] Ortega-Loubon C, Fernández-Molina M, Carrascal-Hinojal Y, Fulquet-Carreras E (2016). Cardiac surgery-associated acute kidney injury. Ann Card Anaesth.

[15] Harky A, Joshi M, Gupta S (2020). Acute kidney injury associated with cardiac surgery: a comprehensive literature review. Braz J Cardiovasc Surg.

[16] Vives M, Wijeysundera D, Marczin N (2014). Cardiac surgery-associated acute kidney injury. Interactive Cardiovasc Thorac Surg.

